# Positive Aspects of Welfare in Sheep: Current Debates and Future Opportunities

**DOI:** 10.3390/ani12233265

**Published:** 2022-11-24

**Authors:** Mukhtar Muhammad, Jessica E. Stokes, Louise Manning

**Affiliations:** 1 Department of Agriculture Food and Environment, Royal Agricultural University, Cirencester GL7 6JS, UK; 2 Lincoln Institute for Agri-Food Technology, University of Lincoln, Riseholme Park, Lincoln LN2 2LG, UK

**Keywords:** positive welfare, sheep, indicators, emotions, affective states, behavior, physiological

## Abstract

**Simple Summary:**

Positive welfare represents an expansion of the traditional animal welfare understanding that animal welfare is defined by minimizing negative experiences such as stress, pain, suffering, and disease. Positive welfare as a concept shifts the narrative from just reducing negative experiences to intentionally providing animals with increased opportunities to have positive experiences and feelings. The concept, although around for several decades, is in its infancy in terms of developing ways of assessing positive welfare on farms. Therefore, the adoption of practices that promote opportunities for positive welfare and experiences faces challenges, especially in monitoring continuous improvement at the farm level. The most apparent challenge is the lack of validated indicators to characterize a full spectrum of positive welfare experiences and feelings for farm animals. Assessing positive welfare in extensively reared animals such as sheep may pose additional practical challenges too. Using an iterative approach, this critical review aims to explore the extent to which positive welfare interventions and indicators are positioned and have been developed within the literature on sheep welfare. This paper critiques existing literature in terms of potential indicators for current and future research and characterizes the practicality and suitability of these indicators for on-farm welfare assessments. Finally, potential aspects of positive welfare for sheep are highlighted so that they may guide future research and practical implementation on farms.

**Abstract:**

The concept of positive welfare is an expansion of the traditional understanding that animal welfare is defined by minimizing stress, pain, suffering, and disease. Positive welfare shifts the animal welfare narrative from a focus on reducing negative experiences to proactively providing animals with opportunities to have positive experiences and feelings. The concept, although around for several decades, is in its infancy in terms of developing ways of assessing positive welfare on farms, especially in extensive systems, and there are challenges in the adoption of positive welfare practices and the monitoring of continuous improvement at the farm level. Using an iterative approach, this critical review aims to explore the extent to which positive welfare interventions and indicators are positioned and have been developed within the animal welfare literature for sheep. This paper critiques existing positive welfare indicators, such as choices in food and the physical environment, conspecific social synchronization, maternal bonds, intergenerational knowledge transfer, positive human–animal relationships, etc., as currently assessed by the ‘good life framework’. It also reviews the characteristics of scientific measures for (positive) affective states in the current sheep literature and their potential contribution to understanding positive welfare states in sheep. In conclusion, this paper provides recommendations for future research regarding sheep welfare.

## 1. Introduction

As animal welfare science develops and there are societal demands for higher standards, the animal welfare narrative has shifted from one of protecting animals’ basic needs to a more holistic understanding of what constitutes quality of life. Initially, the welfare narrative presented that an animal is considered to have a life worth living when all Five Freedoms are satisfied. One of the major limitations of the Five Freedoms is that it only provides a snapshot of the welfare of the animal in its overall quality of life cycle, therefore neglecting other key behavioral issues that the animal learns over the course of its lifespan [1,2]. Moreover, the Five Freedoms emphasized seven affective states as welfare indicators (hunger, thirst, fear, distress, heat stress, physical discomfort, and pain) to alleviate *negative experiences*, with only freedom for natural behavior appearing to promote positive experiences [3]. However, it is now understood that animals, as sentient beings, can experience both positive and negative feelings; therefore, caregivers are responsible for minimizing negative welfare and enhancing positive welfare. In light of this principle, an animal free from pain and suffering but not in an environment that provides positive welfare opportunities may be described as being in a neutral state of welfare. Therefore, more recent animal welfare narratives have built on the Five Freedoms [1,4,5,6].

Positive welfare has emerged as an expanded definition of animal welfare, focusing on what constitutes quality of life or a good life for farm animals. Positive welfare research focuses on creating opportunities for animals to engage in experiences and behaviors that lead to positive welfare states, aiming to increase positive experiences and reduce negative experiences [7,8,9]. The aim of positive welfare is not simply for animals to experience pleasure or feel good, since this is not realistic for animals or humans, but to increase the frequency of positive welfare experiences, thus improving the animal’s quality of life. The good life concept, introduced by the Farm Animal Welfare Council (FAWC), promoted the shift of emphasis for standards for farm animal welfare from a life worth living to a good life [10]. In the absence of suitable positive welfare outcome measures that relate to the animals’ experiences and feelings, the ‘good life’ framework was developed as a resource-based approach that aims to provide positive welfare opportunities that the animal values (above and beyond basic needs), in order for caregivers to facilitate a good life (over and above that of a life worth living) [10,11].

The concept of positive welfare is not only of interest to science but also to society, particularly farmers and the public, who consider both positive and negative aspects of welfare when assessing livestock production systems [12,13,14]. Stakeholders prioritize positive and negative welfare within their given context or situation and this needs to be taken into consideration when developing positive welfare measures. Stokes [14] in participatory research found that dairy farmers support some of the good life opportunities for cattle, while they disagree with other opportunities due to their impracticability at farm level. Bringing all stakeholders together can facilitate common ground for promoting positive welfare through collective decision-making and the implementation of resource-based mechanisms and good practices [13]. Research and reviews have focused on assessing the meanings and assessment of positive welfare in laboratory research animals [15,16,17,18], zoological animals [19,20,21], and aquatic animals [22]. However, there is an inconsistent focus across farm animal species. For example, there is a lack of applied positive welfare assessments developed for sheep compared with other farm animals such as cattle, poultry, and pigs [7,14,23,24,25,26,27]. Therefore, a contemporary overview of studies assessing positive welfare indicators for sheep is warranted, and this critical review aims to answer the following research question:

To what extent are positive welfare indicators articulated, explained, and critiqued within the scientific literature on sheep?

## 2. Materials and Methods

**Figure 1:**
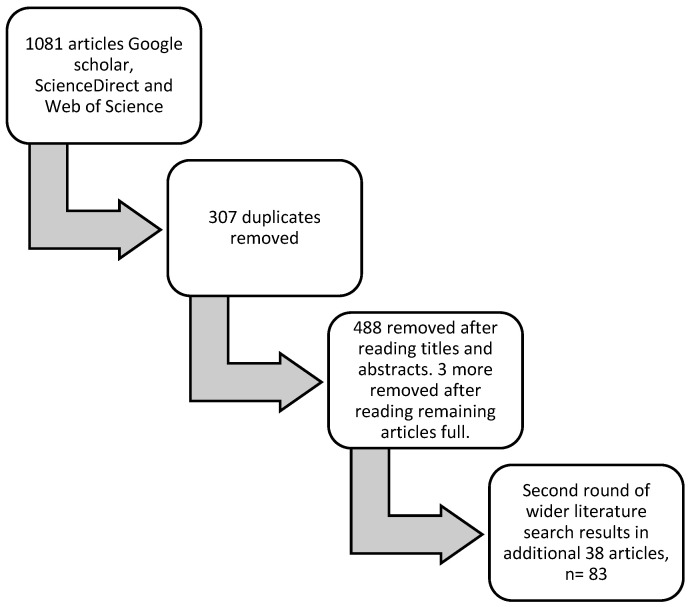
Diagrammatic explanation of review process.

Science Direct, Google Scholar, and Web of Science were used to identify articles for this review, as these are the leading comparable database searches. Google Scholar, for instance, is currently the most comprehensive academic search engine [28], while ScienceDirect and Web of Science appear to have a predisposition for animal welfare science articles [29]. These databases were assessed for literature published between 2009–2022. As alluded to earlier, the concept of positive welfare gained prominence after the FAWC report on the ‘good life’ concept in 2009. Therefore, the scope of the inquiry was set in this timeframe to encompass the development of positive welfare as a concept and positive animal welfare indicators. A structured literature search was undertaken using a series of cyclic/snowballing [30] searches of the terms *indicators*, *positive welfare*, *good life*, *factors*, and *determinants* of sheep welfare. As such, the first 100 items from each result were considered for relevancy and duplication when undertaking the searches for a given combination of search terms. Inclusion criteria included research and review articles relating to mostly describing components of the good life framework, i.e., comfort, pleasure, confidence, and health. Similarly, studies relating to positive welfare outcome assessments were considered. Scientific research on positive welfare for sheep appears to be in its infancy, and, as such, using this first approach, only a limited number of papers were identified. Therefore, another round of literature searches was conducted in a second step, utilizing references cited within relevant articles as well as papers that had been cited within related articles, which were then explored and relevant articles identified. See Figure 1 for the flowchart on the methodology process. In total, we found 83 papers referring to positive welfare, which were read and critiqued and informed the thematic presentation in the next section. In the extant literature, the term measure(s) is used to describe interventions as well as criteria metrics or indicators that can be observed and/or measured scientifically. So, this paper has used the term strategies to describe intentional interventions and then indicators to highlight those criteria that can be observed and/or measured. The works of [31] provided a framework within which this review was structured for resource-based factors associated with positive welfare.

## 3. Result and Analysis

### 3.1. Positive Experiences through Resource-Based Intervention Strategies

The findings have been presented in the following thematic structure for the resource-based interventions and indicators based on an application of the good life concept to sheep which includes: comfort by choice in the physical environment, comfort by choice of the thermal environment while minimizing harm, interest through environmental enrichments, maternal bonding, social synchronization, and confidence derived from the animal–human interaction and promoting positive health.

The positive welfare principle states that where animals are housed in a building throughout the year, they should be loose-housed and at a stocking density that allows them to choose their lying area, so they can all lie synchronously without displacement [31]. Sheep flocks are mostly raised in extensive systems. Providing resources that facilitate comfort and enable sheep to choose where to lie with flock members adds autonomy and improves synchronistic lying without displacements for individual sheep. 

Focusing specifically on comfort and choice, the literature indicates that sheep clearly prefer straw bedding over wooden slats [32]. Furthermore, adding cereal straws to lamb pens during the finishing period and providing ramp play opportunities helps to induce positive stereotypic behaviors and improve the lambs’ adaptation to a novel environment, optimizing their welfare and productivity [33]. The same study also revealed that lambs exposed to straw provisions display foraging behavior and were more active than lambs in the non-provision control. In the same vein, lambs with straw provisions appear to show increased affiliative behaviors, group cohesion, behavioral diversity, and reduced negative stereotypic behaviors [34,35]. In addition, strategies such as the availability of straw bedding improve the comfort of shorn ewes, as reflected in their increased lying time [36]. However, straw provision can be difficult to obtain, and using straw to provide good life opportunities for sheep is cited by some sources as an environmentally unfriendly farm practice [37]. Two alternative enrichments have been explored so far in the literature. Given the choice, sheep appear to choose softer flooring materials, such as mats and woodchips, as demonstrated by their ability to lie on the mats for extended periods compared to the straw [38,39,40,41,42]. The reason for this choice is relatively unclear, and there has been little research to date addressing this crucial gap. Clearly, there is a need for further studies to demonstrate how providing alternate bedding and enrichment resources in the physical environment can, in addition to providing comfort by choice of the physical environment, facilitate positive behavioral outcomes within sheep flocks. Similarly, the sheep literature provides little evidence on the application of neurological and physiological indicators to evaluate the positive welfare states of sheep when provided with these resources. This indicates that the positive welfare research for sheep in this regard is lagging behind other established research in farmed animals such as chickens and pigs [43].

The good life framework holds that sheep should be able to exercise individual preferences for their thermal comfort at all times [31]. Providing access to pasture for housed animals or free movement increases affiliative behaviors and reduces stereotypic behavior in other animals [44,45]. Similarly, when given free access to an outdoor area, sheep prefer to be outside even under extreme conditions [46]. This also strongly suggests that being outside is valuable to them. Furthermore, the shade provided by trees improves sheep’s comfort during extreme summer temperatures [47,48,49]. Sheep that were kept under trees in harsher climates showed normal behavioral characteristics, whereas sheep kept in the open exhibited unnatural behaviors to cope with the stress caused by the heat [47,48,49]. Intervention strategies such as agroforestry or silvopasture, the practice of growing trees within fields, can enhance the quality of life of grazing ruminants with additional medicinal values [50]. The willow tree, for instance, contains anti-inflammatory substances that allow grazing ruminants to ‘self-medicate’ and become more resilient and comfortable within their environment. Therefore, exploring the impacts of such interventions can enhance and contribute to our understanding of resilience (an indicator of positive welfare states) in sheep.

Lying in a ventral posture with legs tucked under can indicate a positive state of calmness in sheep, although this behavior may vary among species, geographical locations [51], or due to parturition [52], suggesting that the reliability of this measure remains imprecise. In addition, the previous study by [52] primarily considered just welfare as a concept and not positive welfare in particular; therefore, additional study needs to consider lying behavior through a positive welfare lens. 

Promoting the positive welfare principle of interest via pasture management and food choice for sheep means that they have the opportunity for choice and autonomy to select what they want to eat. In extensive outdoor systems, animals are free to move within a habitat that allows them to graze on preferred grasses and shrubs. Grazing behavior and forage intake interact strongly with pasture composition diversity and grazing methods. Feeding behavior is shaped not only by the need to maintain the biological functioning of the animal (as determined by body homeostasis) but also by hedonistic behaviors and satiation (post-ingestive effects) [53,54,55]. Satiate effects, or as Mellor [56] calls them, post-consummatory satisfaction, are a component of positive effects derived from past activities (for example, past consumption of a flavored food). Sheep, through reinforcing learning techniques, are able to determine their preferred diet through their use of sensory cues [54,55].

When food is diverse and abundant, satiety for single foods stimulates the exploration and selection of a diverse diet and subsequent food sources [57,58]. Mellor [59] describes such foraging and exploratory behaviors as being accompanied by positive effects mainly due to neuroscience-based evidence. Firstly, the neurological processing of these behaviors includes dopamine neurotransmitters communicating the ‘reward’ signal to the brain of the animal, therefore giving it a sense of satisfaction. Secondly, the affective valence of exploratory behaviors constitutes likes/wants that need to be fulfilled to deliver satiation, and this motivates sheep and their interaction with the environment [59]. In addition, Villalba et al. [57,58] added that herbivores, such as sheep, can also reap the physiological benefits of ingesting compounds with medicinal (i.e., antiparasitic) benefits.

The enriched environment can also stimulate intergenerational knowledge transfer in sheep, where sheep transfer grazing behaviors, especially knowledge of pasture nutrients and palatability, to lambs. The transmission of self-medicative behavior from the ewe to lambs is clear; for example, in one study, ewes could differentiate between polyethylene glycol-rich medicinal compounds to grape pomace and tannin-rich diets [60]. Upon exposure to the same feeds, these experienced ewes and their lambs preferred medicinal plants compared to the inexperienced group. When lambs were isolated from their mothers in both groups and exposed to these feeds, the lambs of experienced ewes were more inclined to consume medicinal plants than those of inexperienced ewes. It is evident from these studies that preference for choice and selection can be passed down through generations of ewes to their offspring. Indeed, such learned patterns of behavior positively influence the lambs’ welfare response by causing beneficial neurological, morphological, and physiological changes in the animals [57].

All these studies infer that animals build interest-related experiences in an enriched environment. Our review suggests the need for up-to-date research on this principle that considers the wider positive experiences and benefits of more species-diverse swards for outdoor sheep. The authors encourage more research into understanding the value of environmental enrichment for sheep and the sheep’s preferences towards it, especially the benefit of shade and micronutrients from the trees in an agroforestry/silvopasture setting. Enriching the pasture-based environment in this way could result in enhanced comfort and interest for the flock and greater pasture and livestock resilience within an existing food production system.

### 3.2. Inter-Animal Interactions

#### 3.2.1. Maternal Bonding

Prosocial behaviors refer to ‘a social act from a donor to a recipient, with the likelihood that this act benefits the recipient, without necessarily precluding benefits to the donor’ [61] (p. 114). This definition distinguishes prosocial behaviors from social behaviors which can be positive or negative. Social behavior benefits those who engage in it, which means that these individuals are better off than they would be on their own or if they had not engaged in this behavior with others [62]. Positive social behaviors refer to all interactions between two or more individuals in a group that, as a result, change the activity in the group and serve as an intra-specific communication method [63]. In contrast, aversive behaviors primarily result in negative emotions such as fear and dread. Five distinct prosocial animal behaviors include caregiving, affiliation, social teaching, sharing, and cooperation [61]. As for prosocial behavior in sheep, parental care through ewe–lamb bonding has been identified as a key aspect for all five behaviors, and social interaction and affiliation within the flock are considered critical factors for positive welfare.

Scientific studies assessing and reviewing ewe–lamb bonding discuss the interconnectedness of physiological, behavioral, and neurological activities that result in filial attachment between ewes and lambs. Nowak et al. [64] explained bonding as a two-way process where the ewe recognizes and displays similar sensory recognition of the lambs. The olfactory bulb around the brain of the ewe undergoes neurophysiological changes during parturition [62,64,65], which could indicate changes in affective states. Additional activations in the hypothalamus and the amygdala also assist the ewe in picking up the amniotic smell of the lambs, leading to the ewe establishing a ‘preference’ for the lamb(s) as being her own [62,64,65,66]. The strong pull towards the lamb enables the ewe to display positive behaviors towards the lamb(s). These include grooming/licking, selective preference, recognition, and seeking proximity [62,65,67]. The lamb in return seeks and singles out the ewe by establishing sucking behaviors. Neurophysiological indicators, involving cholecystokinin and intestinal stimulation, are useful to explain experiences around suckling establishment in lambs [64]. The lambs also display other behaviors such as seeking proximity and responding to separation and union through vocalizations. In other farm animals, low-pitched bleats (vocalizations) can indicate positive experiences, but the same conclusion is yet to be drawn for sheep [68], further highlighting a gap in our understanding of the behavioral–emotional link and associated vocalizations in sheep.

Clearly, the evidence cited has described ewe–lamb bonding as being a positive experience for the ewes without categorically mentioning the ewes in terms of positive welfare states. Thus, more research is required to understand the individual animals’ experiences and their reciprocity of emotions. Furthermore, it would be interesting to observe how ewe–lamb contacts influence the affiliative behaviors of ewes and what the results may imply for our understanding of positive effects in sheep. Studies of this type are already underway in other species [69].

#### 3.2.2. Conspecific Social Synchronization

Social groups are an integral part of the sheep’s complex and dynamic environment, leading to the evolution of many strategies to enhance the group’s survival and sustain its viability. It is known that sheep form social bonds through social hierarchies and have evolved as flock animals living in groups within a close range. Sheep can demonstrate positive (affiliative relationships) and negative personalities (aggressive relationships) in their social environments, and these personalities can influence social learning and cognition [70]. The affiliative personalities are positive, providing opportunities for social support in challenging situations, and are accompanied by calming and rewarding physiological responses [69,70,71,72].

Ozella et al. [73] considered the preferential behavior of sheep using proximity sensors in a way similar to most studies assessing social interactions. They determined that such a relationship occurs between sheep based on their shared, similar attributes. Similarly, the groupings of sheep based on biological sex (ram groups or ewe groups) are believed to drive social cohesion and interaction, and this is also clear in other animals [74]. Nevertheless, Ozella et al. [73], like other studies investigating social interactions, found that genetic relatedness does not appear to influence proximity. One concern is the tendency for proximity to increase when the animals suffer from negative emotions such as fear. In addition, there is a need to investigate thoroughly how familiarity influences affiliation patterns. Moreover, there have been calls to develop alternative indicators of allogrooming in sheep [75], but so far there are limited studies covering this literature gap.

### 3.3. Human–Animal Interactions

#### Confidence Derived from Human–Animal Interaction

The human–animal relationship is one of the most important components of any strategy (including positive welfare) that seeks to enhance the welfare of animals and their stockpersons/caregivers [76]. The human–animal relationship is, in essence, a reciprocal relationship in which the welfare of animals and the reciprocal benefit to humans are intimately interdependent. In some communities, dogs facilitate the interaction between the sheep and the shepherd and are also vital components of sheep production, especially in remote locations. In the UK, for example, shepherds have traditionally used dogs as a handling aid in moving sheep. In other countries where predators are more of a concern, guardian dogs are used that live with the flock. In this context, observations of the sheep and guardian dogs as a strategy against losses to predation are of interest, although dogs, especially unfamiliar dogs, can be a major source of stress for sheep [77]. This stress can directly result from the sheep’s survival instincts to get away from harm, although how the sheep feel towards the dogs they are familiar with remains largely unresearched. Similarly, sheep are likely to be fearful of humans [78]. However, when an animal shows interest in a human, the fear and aversion are attenuated, and the animal may increase its interaction and attachment with the human/shepherd/caregiver. In exchange, the shepherd experiences an internal sense of well-being and satisfaction.

Rault et al. [79] reviewed some interventions and indicators of human–animal relationships that elicit positive experiences in sheep. Two main interventions that were common in the literature concerned tactile contact, e.g., the brushing and stroking of sheep [80,81,82]. Lambs that were fed and stroked appeared to develop an affinity towards the caregiver [80,81,83]. Indeed, lambs that were stroked showed slower heart rates and reflected positive emotional states compared to lambs left unstroked [82]. Tamioso et al. [84] conducted a series of pre-brushing, brushing, and post-brushing phases to observe the behavior of selected sheep. Preliminary results showed that sheep half-closed their eyes in the brushing and post-brushing phases compared with the pre-brushing phase, indicating a relaxation state in the animals. Detailed analysis also revealed that sheep demonstrate confidence and affinity toward the observers by ‘following the observer, leaning against the brush with the head or neck, and stretching the neck when brushed.’ It has also been suggested that the affinity may be conditioned by gentle contact (stroking or brushing) and does not necessarily require food provision or familiarity [81,85].

The feasibility, applicability, and practicality of brushing and stroking sheep on a large-scale farm are challenging. It is impractical for farmers to brush 1000 ewes, as this would be time-consuming. In addition, this activity (brushing) would compete for the farmers’ time with other activities they could be doing to enhance the sheep’s welfare. Moreover, it has been recently demonstrated that additional exposure of lambs to humans in extensive, outdoor settings did not alter their fear of humans [86]. Nevertheless, intervention strategies such as the use of automatic brushing enhance positive behaviors in equines, as indicated by increased positive facial expressions (half-closed eyes, ears turned backward, neck moderately raised) [87], and such interventions can be promoted in sheep when housed. The provision of scratching pads and brushes is now common in housing for cows within a dairy farming system. Similarly, providing shelter in an extensive range for sheep, such as trees or hedging where sheep have the opportunity to scratch, is practical and beneficial.

### 3.4. Positive Healthy Life

#### Building Resilience in Sheep from Breeding Strategies

Resilience is proposed as an indicator of positive health and positive welfare in animals [88]. Resilience links directly with the biological functioning aspects of welfare and describes the normativity of physiological, behavioral, and production performance irrespective of actual production levels, and considering resilience provides insight into the animal’s experience of its environment [88,89]. Therefore, a resilient animal can adapt positively to its environment or maintain or restore good health despite experiencing difficulties coping with short-term adverse environmental factors.

Resilience has been assessed in the literature using phenotypic indicators. Hine et al. [90], while studying the immune response of merino sheep, suggested that selecting for immune competence is feasible and is expected to improve their resilience by enhancing their ability to cope with various disease challenges. Nevertheless, selective breeding also tends to implicate negative as well as positive welfare aspects, and proposals are being made on how to breed efficiently for such desired positive traits [91,92]. In poultry, resilience appears to be affected by both breed and growth rate. Specifically, ‘slow-growing’ broilers were perceived as more resilient than ‘high-growth’ broilers [24]. It will be necessary to undertake empirical research to further develop sheep studies and the associated literature to explore and contextualize animal resilience at the field, flock, and farm levels.

### 3.5. Positive Welfare Assessments

There appear to be a good number of indicators (methods and measures) to assess the affective states of animals (sheep included). However, these methods have not been extensively applied to understand the range of positive welfare states in farmed animals. The main indicators identified included behavioral assessments (ear posture, qualitative behavioral assessment (QBA), play); physiological assessments, including autonomic nervous measures (cardiac response (heart rate), body temperature, respiratory rate and heart rate variability); neuroendocrine measures (including oxytocin, serotonin, dopamine, and opioids); biomarkers (acute phase proteins); cognitive/preference tests (judgment bias, affective bias, and operant condition); and biomedical assessments. These indicators are considered herein in more detail.

#### 3.5.1. Behavioral Assessments

Sheep’s passive ear postures were associated with situations likely to induce positive affective states, such as anticipating feeding [93,94,95,96,97]. Cattle studies observed similar findings [98]. Similarly, passive ear postures occurred when enriched feed was offered compared to wooden pellets [93]. The sheep’s heart rates increased when they received wooden pellets after they expected to be fed enriched feed, suggesting some form of frustration [99]. Ear postures may help assess emotional valence, as shown in cattle, but this needs further investigation in sheep.

An indicator to measure animal emotions from a more holistic perspective is qualitative behavioral assessment (QBA). Wemelsfelder and Mullan [100] provide a very detailed overview of the QBA assessment method. The authors explain it as an approach that captures the expression of the animal as it interacts with the environment. The method is feasible, non-invasive, and easy to use in laboratories, on the farm, during transport, and at marketplaces, as it relies on observers watching and making inferences from the behavioral expression of an animal’s emotional state [100,101,102]. Phythian et al.’s work examines the inter-observer reliability of QBA descriptors for sheep [103]. Evidence from the study shows there were high levels of agreement among assessors based on the duplicate assessment of video footage used in the current study. Another study used a broader group of assessors and supported further exploration of the feasibility and validity of applying this method to sheep welfare assessment [104]. Operant test indicators correlated with QBA in a mixed method approach [105], suggesting reliability in the approach as an assessment method. All these studies indicate that QBA is a valid indicator of positive effects and emotions in animals. However, the subjective elements associated with behavioral observations may impact their perceived validity, especially if there are differences in production systems [51,106]. Thus, further evaluations of the reliability of QBA are necessary to broaden its application to sheep.

The relationship between play and welfare has been explored in several reviews [107,108,109]. The function of play was initially believed to be an expression of animals’ excitement without resulting in any rewards [75]. However, others rejected these thoughts and argued that play is not fully functional but a reinforcing or self-rewarding activity initiated in the context of relative freedom from stress or intense competition [107,108,109]. These contributions in the literature have led to the understanding that play can positively affect animals’ emotions, even if its meaning remains imprecise. Play behavior generates pleasure by activating endogenous opioid systems, regardless of their underlying motivations, which are complex and still not understood [8]. Therefore, an animal playing can indicate the absence of negative emotions and the presence of positive ones. Stress is associated with reduced levels of play, but play can exert a calming effect on farm animals [107,108,109]. Play is associated with the environment where the animal finds itself [107,108,109], and in farm animals it has been categorized as either social, object, or locomotive, where sheep demonstrate all three [110]. Play behavior in sheep has been assessed through expressive behaviors and their duration; for example, lambs exhibit excessive anticipatory behavior towards food when given a choice between food and play, suggesting that food is more critical for lambs than play [110]. Lambs are known to engage in sexual *play* activity in extensive environments; however, ram lambs are more likely to mount than ewe lambs [111]. Similar findings were shown by [112], who revealed time as a crucial parameter for an animal to experience anticipation or frustration.

Play behavior has been proposed as an indicator of the positive affective states of animals [18,109,113]. However, there is still no new information about the quality and intensity of play to make inferences about the influence of a positive affective state on play behavior [18,109]. In addition, earlier studies have stressed the need to investigate how play behaviors differ from lambs to sheep; despite a general interest in such observations, so far, the focus has been limited to sheep studies compared to other species.

In conclusion, the behavioral outcome-based measures reviewed here show potential for assessing positive effects in sheep. Nevertheless, other necessary measures were not identified in this review but are well-established in the literature concerning other species. For example, eye whites are proposed as indicators of positive emotions in cattle, but so far this measure has received less application in sheep. Similarly, behavioral diversity ([19] referenced earlier) is proposed as a potential indicator for positive welfare. With sheep known to have a wide behavioral repertoire, it will be interesting to see if this repertoire can be applied to behavioral diversity analyses for sheep in future research.

#### 3.5.2. Physiological Assessments

Autonomic nervous measures (indicators) have been combined mostly with behavioral assessments to assess affective states in farm animals, including confined sheep and outdoor rearing. Heart rate, body temperature, and blood oxygen and heart rate variability (measured through the root mean square of successive differences, RMMSD) are the most commonly applied autonomic nervous measures of valence in sheep [99,114,115]. Heart rate variability reflects the balances in the sympathetic and parasympathetic autonomic nervous systems [116]. The former reflects low heart rate variability and corresponds to negative valence, while the latter shows higher variability and positive valence [116]. In sheep, assessing heart rate variability has had applications towards studying positive aspects. For instance, Coulon et al. [82] and Reefmann [117] demonstrated that lambs being stroked/groomed by caregivers had higher RMMSD compared to unstroked/ungroomed lambs, indicating the presence of positive experiences. Despite its usefulness in assessing affective elements, heart rate variability results are mixed, like in other species. Other research assessing emotional valence in sheep found that RMMSD shows an inverse pattern, suggesting that heart rate variability is a proxy for the heart rate (cardiac response) measure [99,116]. In any case, the differences in these measures are desirable, especially with a positive welfare lens.

Few studies have explored neuroendocrine measures of effects in the explored literature (oxytocin, dopamine, and serotonin). Oxytocin is a physiological measure that is also reflective of positive welfare states and linked to the mental states of farm animals [118]. Oxytocin hormones are generally released into the brain and blood circulation in response to somatosensory stimulation from touching, nursing, feeding, or other tactile contacts [118,119]. In human–animal bonding, however, it was observed that oxytocin levels did not vary during human contact [119], therefore suggesting that the measure is inconclusive in that regard. Furthermore, Rault et al.’s [118] critical review highlighted the underapplication of oxytocin in the sheep literature, attributed to inconsistent results and a lack of standardization in the approach, suggesting a lack of inter-observer reliability.

As part of their wider study, Wang et al. [120] assessed the neuroendocrine mechanisms involved in regulating maternal behavior. Higher levels of oxytocin, dopamine, norepinephrine, and other neuroendocrine in multiparous ewes suggested they were better maternal caregivers compared to primiparous ewes. In another study, serotonin is suggested to influence the pessimistic-like judgment capabilities of sheep [121], while similar conclusions cannot be drawn for opioids [122]. In the same vein, biomarkers such as acute phase proteins have received little attention in the sheep literature.

In conclusion, research applying neuroendocrine measures in the literature is underrepresented, while autonomous nervous measures require further validation. Future studies are required to assess these affective states of sheep using these neuroendocrine measures, especially from a positive welfare point of view.

#### 3.5.3. Preference Tests

Preference tests are a common and reliable means of assessing the affective states of farm animals [113]. These tests are considered good assessors of the affective states of the animal, explaining pessimistic-like (negative affect) or optimistic-like evaluations of their surroundings [116,123]. In other words, the preference test shows the animal’s affective states through observable behavioral responses. Sheep trained in specific scenarios can then be subjected to tests to choose between ‘this’ or ‘that’ preferred objects, ‘yes or no’ questions, and it can be determined ‘how much’ the animal can ‘work’ towards achieving positive benefits [116]. This openness of the indicators means that they can be adapted to various categories of welfare to measure animal preferences, which has been demonstrated in the literature.

The judgment bias test is considered a good indicator of positive affective states. Sheep rewarded with food had a longer latency to reach the trained location than sheep rewarded with woodchips, indicating the positive impacts of food rewards on their affective states [122]. However, these findings do not distinguish between latency to approach positive cues and latency to approach negative ones. Sheep with positive experiences from enriched housing tended to have shorter latencies to approach ambiguous ‘choice’ positions than standard-housed sheep [124]. Comparatively, sheep from standard housing tended to have shorter latencies to approach food with the novel object present than sheep from enriched housing. These results demonstrate the influence of rewards on positive affective states in sheep. Similarly, when sheep [125] or cattle [126] were released from restraint, they exhibited less anticipatory behavior towards an available reward, as outdoor settings and pastures appeared to provide greater perceived benefits than a known reward. As a whole, preference tests seem to corroborate sheep’s positive affective states and emotions. 

One practical barrier to the broader use of judgment tests in the farm situation is that sheep must be trained for a long time before being tested in the bias test [127]. Performing training can be challenging, inconvenient for researchers, and ineffective in actual-world conditions on farms. As a result, it can be challenging to establish the inter-observer reliability of the measure. Preference tests may also result in animal stress and disengagement when their welfare is not regularly monitored. Farm animals tested frequently may also learn that the tests are not reinforced, leading to changes in their behavioral responses [127]. Affect-driven attention bias that does not require training animals has been suggested as an alternative to judgment bias [127]. Affect-driven tests are the main approaches to measuring moods in sheep, corroborating earlier findings on other species. Sheep clearly showed their dislike for negative stimuli (pricking), intermediate (slight pressure), and positive stimuli (kneading) [128]. The intermediate stimuli, however, may reflect a point of balance (where the animal does not *consciously* feel pain: see Mellor [129] for more on this); however, this is not clearly defined in the referenced literature. Therefore, the results of the study do not clearly show whether sheep were able to differentiate between intermediate and positive stimuli.

There are suggestions that affect-driven bias tends to focus on negative rather than positive effects [130,131]. In addition, attention bias may involve pharmacological interventions, whose role in inducing affective states remains unclear in sheep research [132,133,134]. The inconsistencies in these results showed that there are still gaps in our understanding of positive valence in sheep. Nevertheless, utilizing this measure (affective tests) could add value to existing preference testing. As the welfare science community learns more about measuring the cognitive abilities of sheep, these tests can start to be used to measure the extent to which resources in their environment are facilitating positive affective states.

The behavioral demand models are established indicators commonly applied for assessing motivations in preference testing. This application has been used in conjunction with the approach-avoidance preference tests to determine affective valence through positive behavioral engagement (repeatedly going back to the cue/video) or negative behavioral disengagement (the animal does not like the video) [135]. Raoult et al. [135] demonstrated that sheep tend to avoid videos of negative stimuli (moving dogs) when subjected to the operant conditioning method compared to other preference tests. Earlier, Verbeek et al. [136] measured feeding motivation in sheep using the operant condition method. Sheep with lower body condition scores were more motivated to reach feeding positions, despite the ‘cost’ of walking, compared to sheep with a higher body condition score. Similar results were observed in [137,138], the latter of which appraised multiple behavioral demand models in their study. All these studies have, however, primarily focused on negative affective assessments. Future assessments of the sheep behavioral preferences using operant conditioning are therefore desirable from a positive affective states angle.

In any case, there is a need for more research to ensure that measures under preference tests are reliable and consistent in their applicability to assessing positive effects in sheep.

#### 3.5.4. Biomedical Techniques

Biomedical techniques are used in research to assess animal mental states, behavior, and emotions [113]. They include electroencephalography (EEG), optical imaging, fMRI, and positron emission tomography [113]. The EEG spectrum analysis has been used to study lateralized behaviors related to brain function asymmetries in farm animals. For example, equine studies recently revealed negative correlations between the left hemisphere and stereotypic behaviors, suggesting that the brain’s left hemisphere is responsible for a positive welfare state [139]. Similar hypotheses are buttressed by other findings in other species, as reviewed by [140], but not recently for sheep. Successful applications of EEG techniques have been used to assess negative affective states in sheep [141]. Similarly, functional near-infrared spectroscopy (fNIRS) was used to assess positive effects in sheep [142], but inconclusive results mean that repeatability studies are required, and this has not been addressed so far. Altogether, there is limited available information on biomedical measures (indicators) with a positive welfare application.

## 4. Conclusions

The purpose of this critical review was to draw together extant literature to then discuss and critique the extent to which positive welfare interventions and indicators are positioned and have evolved in the literature on sheep. The aim was to answer the research question:

To what extent are positive welfare indicators articulated, explained, and critiqued within the scientific literature on sheep?

As part of the study, the literature on resource-based, outcome-based interventions and indicators was examined to demonstrate what sheep want to experience to enhance their quality of life when given a choice: this being a fundamental principle of the good life framework. Our findings support earlier research in that much of the current focus originates from the animal’s likes and wants and associated positive outcomes [4]. Behavioral assessments appear to dominate research compared to physiological approaches in terms of outcomes. This is likely due to relative practicality, as well as Lawrence et al.’s [7] indication that behavioral approaches tend to be favored because they are the ‘closest proxies to positive affective states and motivations that ultimately matter for the animal’. However, it is widely discussed that behavioral approaches alone tend to be problematic and may require insights into the biological basis of emotions and the mechanisms of learning and memory, certainly at the stage of validating potential behavioral indicators (measures) for application on farms as positive welfare assessments. This review identified a number of positive welfare strategies, i.e., opportunities facilitated by providing sheep with experiences, resources, and environments that they value. Therefore, using strategies through interventions and associated assessment indicators both with a positive welfare lens is desirable.

There are, however, some principles of positive welfare that are currently underrepresented in the sheep literature. This is a particular gap as scientists and practitioners consider the impact of climate change on extensively reared animals such as sheep in terms of their resilience as animals, the physical environment, access to resources, and the need for shelter as well as more diverse food choices which benefit both the animal and environment. Collaboration between stakeholders and sharing ways in which positive welfare experiences can be facilitated on farms is essential to understanding what practices, interventions and indicators (measures), and situations they consider relevant to enhance the quality of life of sheep in both housed and extensive environments. As there is a lack of application of these welfare concepts on farms, stakeholders must work together to frame how they provide the resources, experiences, and environment for a positive outcome for sheep, to ensure the uptake of measuring the positive impact of these interventions reliably. Working together to improve the reliability and relevancy of positive welfare interventions and indicators will also aid transparency and clarity in communication with multiple key stakeholders, especially consumers, and in turn deliver positive welfare as a social good to society.

## Data Availability

Not applicable.
